# Insulinome de haut grade découvert après 5 ans d’hypoglycémies graves

**DOI:** 10.11604/pamj.2017.27.250.13144

**Published:** 2017-08-04

**Authors:** Nadia Anoun, Hamza Hasnaoui, Firdaous Ameziane Hassani, Mehdi Bourakkadi Idrissi, Abdelmalek Ousadden, Hanan El Ouahabi, Khalid Ait Taleb

**Affiliations:** 1 Service d’Endocrinologie, Diabétologie et Nutrition, CHU Hassan II, Fès, Maroc; 2 Service de Chirurgie Viscérale A, CHU Hassan II, Fès, Maroc; 3 Faculté de Médecine et de Pharmacie – Université Sidi Mohammed Ben Abdellah de Fès, Maroc

**Keywords:** Hypoglycémies, insulinome, open-coelioscopie, Hypoglycaemias, insulinoma, open laparoscopy

## Abstract

Les tumeurs endocrines fonctionnelles du pancréas sont des lésions rares, parmi lesquelles les insulinomes sont les plus fréquents. La majorité des patients atteints d'insulinome ont entre 30 et 60 ans et 59% d'entre eux sont des femmes. La plupart des insulinomes sont sporadiques, alors que 10% sont développés chez des patients atteints de NEM. Le diagnostic repose sur la clinique et sur le bilan biologique. Le bilan d'imagerie préopératoire (Echo-endoscopie, TDM, IRM) est essentiel localisant la tumeur dans plus de 80% des cas. Le traitement de référence est l'exérèse chirurgicale de la tumeur. Nous rapportons ici un cas d'insulinome de haut grade développé sur la face antérieure de la queue du pancréas chez une patiente de 50 ans.

## Abstract (en)

Functional endocrine tumors of the pancreas are rare. Among them insulinomas are the most common types. The majority of the patients with insulinoma are between 30 and 60 years of age and 59% of them are women. Most insulinomas are sporadic tumors while 10% of them develop in patients with multiple endocrine neoplasia (MEN). Diagnosis is based on clinical examination and laboratory tests. Preoperative imaging assessment (echo-endoscopy, CT, MRI) is essential for tumor localization in more than 80% of cases. Surgical excision is the treatment of choice. We here report the case of a 50-year old patient with high grade insulinoma developed on the anterior surface of the pancreas tail.

## Introduction

Les tumeurs neuroendocrines digestives représentent un groupe hétérogène de tumeurs développées aux dépens des cellules du système endocrinien diffus, parmi lesquelles les insulinomes sont les plus fréquents [1]. De siège presque exclusivement pancréatique, l'insulinome est bénin dans 90% des cas [2]. Le diagnostic positif est clinico-biologique. Le bilan topographique est nécessaire pour guider le geste thérapeutique [3,4]. Nous rapportons un cas d'insulinome de haut grade siégeant au niveau de la queue du pancréas.

## Patient et observation

**Figure 1:**
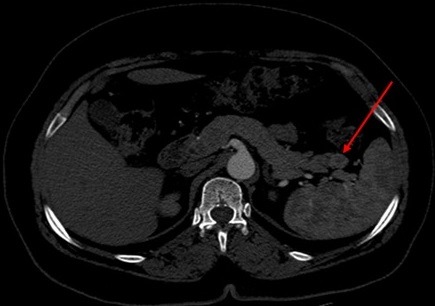
TDM abdominale en coupe axiale C+: lésion de la queue du pancréas se rehaussant après injection du produit de contraste

**Figure 2:**
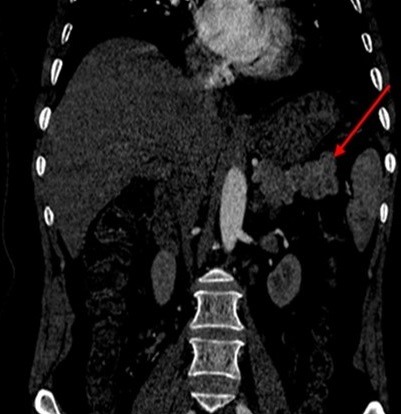
TDM abdominale en reconstruction coronale montrant une lésion de la queue du pancréas mesurant 1,5 cm de grand axe

**Figure 3:**
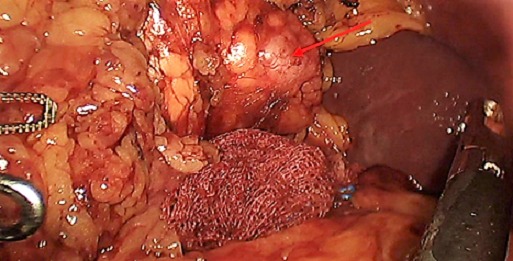
Lésion de 1.5 cm de grand axe de la queue du pancréas correspondant à la lésion décrite radiologiquement

**Figure 4:**
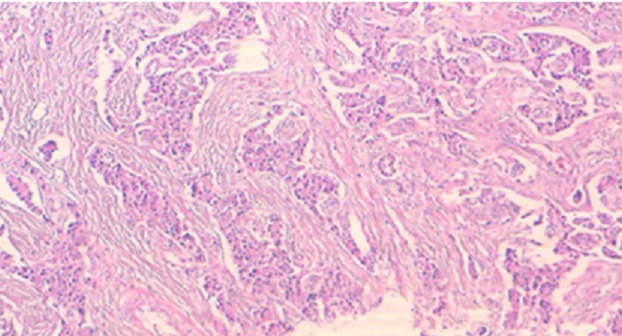
Prolifération tumorale infiltrant le parenchyme pancréatique, disposée en nids et en massifs; les cellules tumorales sont monomorphes, le noyau est discrètement irrégulier avec un fin nucléole, le cytoplasme est abondant et éosinophile

**Figure 5:**
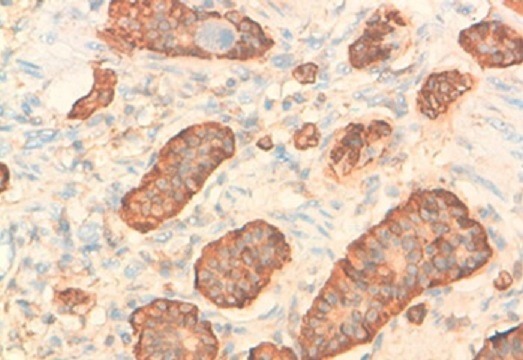
Expression cytoplasmique et granulaire de la chromogranine A et de la synaptophysine par les cellules tumorales

Il s'agit d'une patiente de 50 ans, sans antécédent pathologique notable, qui présente depuis 5 ans des malaises hypoglycémiques précédés de signes neurovégétatifs à type de flou visuel, troubles de conscience pouvant aller jusqu'au coma hypoglycémique, survenant à jeun et à distance des repas, sans rapport avec l'effort, devenant plus sévères et plus fréquents, à raison de 2 à 3 épisodes/jr, cédant au resucrage oral, confirmés par une glycémie à jeun veineuse à 0,26 g/l, avec notion de prise pondérale non chiffrée durant ces 5 ans. La recherche biologique initiale a révélé une insuffisance surrénalienne devant un cortisol plasmatique de 8h à 5 µg/dl (14-20), mise sous substitution en hydrocortisone avec persistance des hypoglycémies. Devant la triade de Whipple, un insulinome fut évoqué, confirmé par le profil biologique d'une sécrétion inappropriée d'insuline (65,9 mUI/l (2,6-24,9) et de Peptide C (9,2ng/ml (1,1-4,4). Dans le cadre du bilan topographique, une echo-endoscopie a été initialement réalisée. Elle était sans particularité, complétée par une tomodensitométrie abdominale en coupes fines avec injection du produit de contraste révélant une lésion nodulaire caudale du pancréas, réhaussée après contraste, faisant 1,5 cm de grand axe (Figure 1, Figure 2). Par ailleurs, le bilan à la recherche d'un néoplasie endocrinienne multiple de type 1 est revenu négatif. La patiente a bénéficié d'une pancréatectomie caudale sans splénectomie par open-coelioscopie abdominale (Figure 3). Les suites post-opératoires ont été marquées par la formation d'un faux kyste péri-pancréatique pour lequel une surveillance a été préconisée, avec une disparition totale des hypoglycémies. L'étude anatomopathologique de la pièce opératoire a confirmé la nature neuroendocrine de la tumeur. Elle était bien différenciée, classée grade 2 selon la classification de l'OMS 2010, infiltrant la graisse péri-pancréatique, avec présence d'emboles vasculaires et d'un engainement péri-nerveux. Les limites de résection étaient saines (Figure 4, Figure 5).

## Discussion

L'insulinome est une tumeur rare puisque la plupart des séries rapportent une incidence de 0,5 à un cas par million de patients par an [5]. Il constitue cependant la plus fréquente des tumeurs endocrines pancréatiques. Il est le plus souvent unique et bénin, de taille inférieure à 2 cm, la forme maligne est beaucoup plus rare et retrouvée dans seulement 5 à 11% des cas [6]. Dans 5 à 10% des cas, l'insulinome s'intègre dans le cadre des néoplasies endocriniennes multiples de type 1, associant des tumeurs endocrines du pancréas, de la parathyroide, de l'antéhypophyse, de la corticosurrénale, du thymus ou des bronches [6]. Aucune association de ce genre n'a été constatée chez notre patiente. L'âge moyen de survenue de l'insulinome est de 52 ans avec une prédominance féminine [5]. Il est exceptionnellement rapporté chez le sujet âgé et l'enfant. Le délai diagnostique est relativement long, en raison de l'absence de spécificité clinique, d'environ 12 à 18 mois en moyenne [7], arrivant à 5 ans chez notre patiente. Les symptômes, traduisant une hypoglycémie, surviennent notamment à jeun et à distance des repas, associant asthénie, flou visuel, vertige, céphalées, sueurs froides pouvant aller à des troubles de conscience. Le tableau clinique de notre patiente était fait typiquement d'une triade de Whipple. Le diagnostic biologique de l'insulinome est facilement posé devant l'association d'une hypoglycémie à un taux élevé d'insulinémie et de peptide C. Dans les cas douteux, une épreuve de jeune prolongée jusqu'à 72h peut être proposée. Le diagnostic topographique se confronte à de nombreuses difficultés, la tumeur étant en général de petite taille, mesurant moins de 1 cm dans 24% des cas, et de 1 à 2 cm dans 42% des cas [8]. Il est néanmoins nécessaire, avant de se lancer dans une chirurgie difficile, de pouvoir localiser la tumeur et de pouvoir affirmer qu'elle est unique. L'intérêt du repérage grâce à l'imagerie non invasive (échographie, scanner) reste discuté.

Cependant, les progrès récents du scanner (multibarrettes en coupes minces), de l'imagerie par résonance magnétique, les excellents résultats de l'échoendoscopie et de la scintigraphie à la somatostatine (octréoscan), ont fourni un regain d'intérêt au repérage préopératoire de l'insulinome. Toutefois, deux examens dominent cette étape : l'échoendoscopie peut mettre en évidence de petites lésions nettement infracentimétriques dans le pancréas, voire millimétriques dans la paroi duodénale. Dans les rares cas d'insulinome malin, l'échoendoscopie peut mettre en évidence des adénopathies péripancréatiques permettant d'orienter d'emblée le geste à visée carcinologique. Chez notre patiente, la tumeur n'a pas été visualisée à l'echo-endoscopie, ceci peut être expliqué par la localisation dans la queue du pancréas qui rend plus difficile l'identification de la lésion à l'échoendoscopie [9]; le scanner en coupe fine du pancréas, avec coupes en phase artérielle précoce, dont la sensibilité atteint 80% selon les dernières études [1, 4], et qui nous a permit de localiser la lésion chez notre patiente. Avant la prise en charge thérapeutique d'une tumeur endocrine du pancréas, la recherche d'une polyendocrinopathie, type NEM I, est un préambule indispensable, puisque son identification amènerait dans tous les cas à une stratégie chirurgicale différente [10]. Il n'existe pas de marqueur biochimique spécifique de la NEM I comme l'est la calcitonine dans les NEM II. Cependant, les antécédents familiaux, la multifocalité des tumeurs endocrines et surtout la mise en évidence d'une hyperparathyroïdie, presque toujours préexistante même si elle est latente, grâce au dosage de calcémie corrélé au taux de parathormone intact, permettent d'affirmer le diagnostic [11]. L'objectif thérapeutique est double: le contrôle des sécrétions hormonales et l'exérèse tumorale [12]. En cas d'insulinome, le diazoxide (per os, 150-600 mg/j) est le plus souvent suffisant en préopératoire. Dans les formes avancées ou en cas d'intolérance au diazoxide (œdème résistant aux diurétiques thiazidiques, hirsutisme, nausées), l'évérolimus (5-10 mg/j) [13] ou les analogues de la somatostatine sont utilisés en débutant par une forme sous-cutanée [14]. Le contrôle sécrétoire des formes avancées est obtenu dans environ 50 % des cas d'insulinomes sous diazoxide ou analogues de la somatostatine. De ce fait, en cas d'insulinome, le recours à la chirurgie et/ou la réduction tumorale sont systématiquement proposés [15].

L'abord chirurgical classique d'une tumeur endocrine du pancréas nécessite une large voie d'abord afin de pouvoir explorer le bloc duodénopancréatique et la totalité de la glande. En pratique, l'intervention comporte soit une médiane sus-ombilicale débordant de quelques centimètres l'ombilic vers le bas, soit une grande incision bi-sous-costale donnant un accès particulièrement étendu sur la région en cas d'obésité, ce qui est un phénomène fréquent chez les patients porteurs d'insulinome, compensant leur hypoglycémie par des apports alimentaires répétés. Après l'exploration visuelle et palpatoire de la glande, l'échographie peropératoire est réalisée. Elle permet le plus souvent de confirmer les données de l'exploration conventionnelle, parfois de déceler une lésion passée jusqu'alors inaperçue (10 % des cas) ou de petites lésions multiples méconnues, profondément enchâssées dans le parenchyme et permettant de porter le diagnostic de NEM I. En cas d'adénome superficiel de petite taille (moins de 2 cm), non adhérent au Wirsung (données échographiques), l'énucléation est l'intervention de choix, qui permet au mieux de ménager le parenchyme pancréatique et d'éviter le diabète postopératoire [16]. L'hémostase de la loge d'énucléation est effectuée progressivement par électrocoagulation bipolaire ou par utilisation de petits clips. Après énucléation, l'exploration de la loge est effectuée de façon minutieuse à la recherche d'une brèche du Wirsung ou d'une fuite du liquide pancréatique. Toute l'intervention doit être réalisée, depuis l'induction anesthésique, sous monitorage de la glycémie dans le sang périphérique toutes les 10 minutes. Cette surveillance constante met le patient à l'abri de toute neuroglycopénie sans traduction clinique sous anesthésie générale, qui pourrait aboutir à des dommages cérébraux irréversibles. L'apport de glucose par les perfusions est arrêté immédiatement après l'ablation de la tumeur, mais la remontée franche de la glycémie peut être différée. Dès le début de l'intervention, des prélèvements périphériques d'insuline sont réalisés. Ils sont répétés 15 à 20 minutes après exérèse. L'insulinémie devrait être redevenue à la normale, sa demi-vie étant de 5 minutes [17].

Sur le plan anatomopathologique, les insulinomes n'ont pas de caractéristiques cytoarchitecturales permettant de les différencier des autres tumeurs neuroendocrines. L'identification des formes malignes n'est pas toujours facile et repose sur les critères anatomopathologiques établis par l'OMS en 2010 [18]. L'insulinome est considéré à pronostic incertain si présence d'un des critères anatomopathologiques suivants: taille supérieure à 2 centimètres ou de grade 2 d'après la classification de l'OMS 2010 ou invasion vasculaire et/ou péri-nerveuse ou présence de nécrose. Il est considéré comme bénin si absence des caractéristiques précédentes. La malignité de l'insulinome est affirmée pour les tumeurs classées grade 3 de la classification de l'OMS 2010 ou par la mise en évidence d'une rechute, d'une extension tumorale locorégionale extra-pancréatique ou ganglionnaire ou à distance. En cas d'insulinome classé bénin, opéré avec une résection R0, aucune surveillance n'est proposée. En cas d'insulinome classé de pronostic incertain selon l'OMS 2010, bien que l'intérêt de la surveillance ne soit pas démontré, les dernières études proposent de réaliser 2 bilans (examen clinique et IRM abdominale) à 6 mois puis annuellement pendant 5 à 10 ans; puis, tous les 2 à 5 ans à vie. L'intérêt de cette stratégie devra faire l'objet d'une nouvelle analyse après obtention d'une cohorte suffisante de patients suivis. Cette stratégie est notamment à proposer pour les exérèses incomplètes R1. En cas d'insulinome malin, deux bilans cliniques et morphologiques à 3 mois sont réalisés puis en cas de stabilité, répétés tous les 3 à 6 mois [19].

## Conclusion

Le diagnostic d'insulinome est clinico-biologique. La localisation de la tumeur reste l'étape la plus difficile. Le traitement de référence est l'énucléation par laparotomie ou laparoscopie. Les résections pancréatiques sont résérvées aux insulinomes malins ou s'intégrants dans le cadre d'une NEM. La survie à 5 ans des insulinomes est de 97% et les facteurs de mauvais pronostiques sont la malignité et l'association à une NEM.

## Conflits d’intérêts

Les auteurs ne déclarent aucun conflit d'intérêts.
